# Significance of the *parkin *and *PINK1 *gene in Jordanian families with incidences of young-onset and juvenile parkinsonism

**DOI:** 10.1186/1471-2377-8-47

**Published:** 2008-12-16

**Authors:** Ronny Myhre, Stina Steinkjer, Alice Stormyr, Gina L Nilsen, Hiba Abu Zayyad, Khalid Horany, Mohamad K Nusier, Helge Klungland

**Affiliations:** 1 Department of Laboratory Medicine, Children's and Women's Health, Faculty of Medicine, Norwegian University of Science and Technology, N-7006 Trondheim, Norway; 2 Department of Biochemistry and Molecular Biology, Faculty of Medicine, Jordan University of Science and Technology, Irbid 22110, Jordan; 3 Department of Neurology, King Hussein Medical Centre, Royal Medical Services, Amman 11821, Jordan

## Abstract

**Background:**

Parkinson's disease is a progressive neurodegenerative disorder, where most cases are sporadic with a late onset. In rare incidences familial forms of early-onset parkinsonism occur, and when recessively inherited, cases are often explained by mutations in either the *parkin *(PARK2) or *PINK1 *(PARK6) gene or on exceptional occasions the *DJ-1 *(PARK7) or *ATP13A2 *(PARK9) gene. Recessively inherited deletions/duplications and point mutations in the *parkin *gene are the most common cause of early-onset parkinsonism known so far, but in an increasing number of studies, genetic variations in the serine/threonine kinase domain of the *PINK1 *gene are found to explain early-onset parkinsonism.

**Methods:**

In this study all families were from a population with a high incidence of consanguinity. We investigated 11 consanguineous families comprising 17 affected with recessively inherited young-onset parkinsonism for mutations both in the *parkin *and *PINK1 *gene. Exons and flanking regions were sequenced, and segregation patterns of genetic variation were assessed in members of the respective families. An exon dosage analysis was performed for all exons in both genes.

**Results:**

In the *parkin *gene, a three generation family was identified with an exon 4 deletion segregating with disease. Both affected were homozygous for the deletion that segregated on a haplotype that spanned the gene in a haplotype segregation analysis that was performed using additional markers. Exon dosage analysis confirmed the recessive pattern of inheritance with heterozygous deletions segregating in healthy family members. In the *PINK1 *gene we identified two novel putative pathogenic substitutions, P416R and S419P, located in a conserved motif of the serine/threonine kinase domain. Both substitutions segregated with disease in agreement with a recessive pattern of inheritance within respective families and both were present as homozygous in two affected each. We also discuss common polymorphisms in the two genes found to be co-segregating within families.

**Conclusion:**

Our results further extend on the involvement of *PINK1 *mutations in recessive early-onset parkinsonism with clinical features similar to carriers of *parkin *mutations.

## Background

Parkinson's disease (PD) is a neurodegenerative disorder recognised by a combination of the different motor symptoms rigidity, tremor, postural instability and bradykinesia [1,2]. Although the onset of PD is, in most cases, considered to be sporadic, individuals with a first-degree relative with PD are at greater risk of developing disease. According to familial aggregation studies the risk of developing PD for first-degree relatives is 2–14 times higher than for those with no family history of PD with relatives of late-onset at the lower and early-onset at the higher end of the range [3-5].

Only a few genes are known to be recessively inherited in early-onset parkinsonism. These genes are *parkin *[6], *PINK1 *[7], *DJ-1 *[8] and *ATP13A2 *[9]. The most common form of early-onset parkinsonism known so far is autosomal recessive juvenile parkinsonism (AR-JP), caused by loss-of-function mutations in the *parkin *gene [10]. AR-JP was first discovered and described in Japanese families [11-14] and subsequently linked to 6q25.2-27 [PARK2; OMIM 600116] [15]. The *parkin *gene was characterized by Kitada *et al*. [6], and has four motifs; an ubiquitin-like domain in the amino-terminal and a RING1-in-between-RINGS (IBR)-RING2 structure in the carboxyl terminal [16]. This characteristic structure links the Parkin protein to the ubiquitin-proteasome system [6].

Different classes of mutations have been described in the *parkin *gene [6] and recent studies have identified several deletions, duplications and point mutations in individuals with this form of parkinsonism [17-20] estimated to be responsible for 50% of autosomal recessive early-onset and 18–20% of sporadic early-onset cases of parkinsonism [10,21]. A possible role for autosomal dominant or compound heterozygous effects of mutations has also been suggested [22].

*PINK1 *was identified as the gene associated to the PARK6 candidate region of PD, mapped to 1p36 [PARK6; OMIM 605909] by Valente and colleagues [7,23,24]. The gene encodes a PTEN-induced putative kinase1 (PINK1) with a serine/threonine kinase motif and a mitochondrial targeting sequence [7]. Recently, several mutations in *PINK1 *have been recognised as disease causative in a growing number of families with early-onset autosomal recessive parkinsonism [25-30], and in sporadic early-onset parkinsonism [31]. The effect of heterozygous mutations and a possible existence of gain of function mutations with dominant transmission has been discussed [32].

Most PD associated mutations have been identified in the serine/threonine kinase domain potentially affecting the kinase activity or possibly the substrate binding ability [7,25-27,29,32-38]. The serine/threonine kinase activity of this segment was confirmed by Sim and colleagues [33] using the kinase domain and C-terminal in truncation experiments. Cell culture studies identified a mitochondrial localisation and function of PINK1 [39].

Clinically, patients with *parkin *mutations are observed as levodopa-responsive parkinsonism [41,42] with a relatively long duration and slow progression [40,41]. As a generalization, the common symptoms of *PINK1 *mutation carriers are clinically described as comparative to symptoms of sporadic PD except for earlier onset and slower disease progression [28,30,34], however, sometimes without dystonia and sleep benefit, which is common for carriers of *parkin *mutations [7,10].

We examined families from Jordan with a high incidence of consanguineous marriages, a recessive pattern of inheritance, and at least one patient with parkinsonism in each family to investigate the significance of *parkin *and *PINK1 *mutations in the region. In light of the ongoing discussion regarding the impact of heterozygous mutations in both *parkin *and *PINK1*, and to discuss a possible effect of mutational load, we present nearly full distribution of substitutions observed.

## Methods

### Patients

This study included 11 consanguineous families with 1–2 affected with juvenile or young-onset parkinsonism with a total of 59 family members with 17 affected. Five families had only one affected member strictly classified as having young-onset sporadic parkinsonism. The age at onset was on average 26.8 ± 5.8 years [range 19–36] covering cases defined as either young (≥ 21 and <40) or juvenile (<21) onset [43,44].

The parents were unaffected with the absence of disease in several generations suggesting an autosomal recessive mode of inheritance. Clinical manifestations included marked response to levodopa (60%), sleep benefit (95%), observed L-dopa induced dyskinesia (90%), dystonic posture (90%), and a slow disease progression (90%). Families A, B, C, D, E and K were inhabitants of the same geographic region in north-Jordan while families F, G, H, I and J were inhabitants at different locations of mid-Jordan.

### Genetic studies

DNA was collected from all available family members. Blood samples were collected on EDTA tubes after gathering informed consent from each participant in compliance with the Helsinki Declaration and the project was approved by the research ethics committee for research on human, headed by the dean of Medical School Jordan. DNA was extracted using Wizard^® ^DNA Extraction kit (Promega). All DNA samples were amplified using GenomiPhi DNA Amplification Kit (Amersham Bioscience Corp) due to limited amounts of DNA. Verification of family relations were performed with the AmpFlSTR^® ^profiler^® ^kit (Applied Biosystems, Foster City, CA, USA) according to the manufacturer's protocol.

Coding regions of *parkin *(exons 1–12, primers available on request) and *PINK1 *(exons 1–8) were sequenced using primers from Hatano et al [25] and additional primers for exon 5 from Schlitter et al [45]. DNA amplification was performed with 10 μM of each primer, GeneAmp 10× buffer II (Applied Biosystems), 25 μM MgCl_2_, 10 μM dNTP and Taq Polymerase (250 Units, 5 U/μl). The PCR was run for 45 cycles at 96°C for 30 sec, annealing temperature for 30 sec and 72°C for 30 sec. Sequencing reactions were performed with BigDye Terminator v3.1 Cycle Sequencing Kit (Applied Biosystems) according to manufacturers manual using 3,2 pmol primer and 30–90 ng DNA template, run for 25 cycles at 96°C for 10 sec, annealing temperature for 5 sec and 60°C for 4 min. Special PCR procedure was used for the GC rich exon 1 (method available on request).

To perform haplotype segregation analysis we genotyped four microsatellite markers (D6S1599, D6S305, D6S411 and D6S1550) frequently analysed in *parkin*. The markers were amplified using 10 μM of each primer, GeneAmp 10× buffer II (Applied Biosystems), 25 μM MgCl_2_, 10 mM dNTP and Taq polymerase Gold (250 units, 5 U/μl). The PCR was run for 40 cycles at 96°C for 30 sec, annealing temperature for 30 sec and 72°C for 30 sec.

To map the deletion size, PCR reactions were performed of ~200 bp fragments at increasingly closer intervals to the breakpoints according to the following conditions; 10 μM of each primer, GeneAmp 10× buffer II (Applied Biosystems), 25 μM MgCl_2_, 10 mM dNTP and Taq polymerase Gold (250 units, 5 U/μl). The PCR was run for 45 cycles at 96°C for 30 sec, annealing temperature for 30 sec and 72°C for 30 sec.

Multiple sequence alignment was performed using ClustalW software available at the European Bioinformatics Institute  to evaluate conservation of amino acids in the *parkin *and *PINK1 *gene across species. Tolerance of the substitutions observed was predicted using SIFT v.2 . Gene exon dosage analysis was performed using the SALSA MLPA P051/P052B kit (MRC-Holland) according to the manufacturer's protocol. Fragments were analysed using GeneMapper v3.7 (Applied Biosystems) and interpretation of results was performed with the Coffalyser v4 software package (MRC-Holland). When not specified in method, fragments and sequences were analysed on an ABI 3130xl Genetic Analyzer (Applied Biosystems, Foster City, CA, USA)

## Results

**Figure 1:**
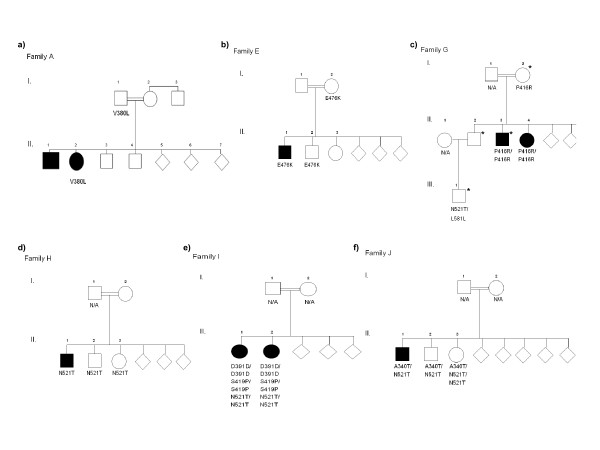
**a-f: Family pedigrees with segregation patterns of substitutions identified in Jordanian families with young-onset parkinsonism**. c The P416R mutation is present as homozygous in both affected of family G. An asterisk shows *parkin *Q34R segregation. e In family I the S419P mutation is present in homozygous state in both affected together with the putative polymorphisms D391D and N521. a-f Substitutions presented in the pedigrees. The A340T, E476K and N521T substitutions could be discussed, regarding mutational load and effect on disease development. An accumulation of substitutions are observed in some of the pedigrees. (N/A -Not available for DNA analysis).

In Family F, a three generational pedigree we identified an exon 4 deletion in the *parkin *gene and substitutions in the *PINK1 *gene co-segregating with disease making an interesting contribution to the discussion on possible digenic effects. Additionally, two non-synonymous substitutions and one synonymous substitution was observed in the *parkin *gene (figure 1a,c). Five non-synonymous substitutions and three synonymous substitutions were identified and among these, two novel substitutions, P416R and S419P, are proposed to be pathogenic mutations. In six families there were carriers of one or more of the *PINK1 *non-synonymous substitutions. In four of these families, substitutions were present as homozygous or compound heterozygous in affected individuals where substitutions segregated with disease in a pattern compatible with recessive inheritance (figure 1b–f) (Overview Table 1).

**Table 1:** Synonymous and non-synonymous substitutions found in the *parkin *and *PINK1 *gene in affected with parkinsonism.

**Family**	**Affected**	**Substitutions in Gene**		**Gender**	**Age at onset**
		***Parkin***	***PINK1***		
Family A	II.1	ND	ND	Male	30
	II.2	V380L (He)	ND	Female	28
Family B	II.1	ND	L63L (He)	Female	23
Family C	II.1	ND	ND	Female	20
Family D	II.1	ND	ND	Female	25
	II.2	ND	L63L (He)	Female	20
Family E	II.1	ND	E476K (He)	Male	20
Family F	II.2	**Exon4 Δ **(H)	E476K, N521T (CH)	Male	32
	II.4	**Exon4 Δ **(H)	E476K, N521T (CH)	Male	32
Family G	II.2	ND	**P416R **(H)	Male	23
	II.3	V17V, Q34R (CH)	**P416R **(H)	Female	25
Family H	II.1	ND	N521T (He)	Male	19
Family I	II.1	ND	D391D, **S419P**, N521T (all H)	Female	36
	II.2	ND	D391D, **S419P**, N521T (all H)	Female	35
Family J	II.1	ND	A340S, N521T (CH)	Male	36
Family K	II.1	ND	L63L	Female	25
	II.2	ND	L63L	Male	27

Putative pathogenic mutations are in bold. CH – compound heterozygous; H – Homozygous; He – Heterozygous; Δ – deletion; ND – none detected.

### Non-synonymous substitutions

**Figure 2:**
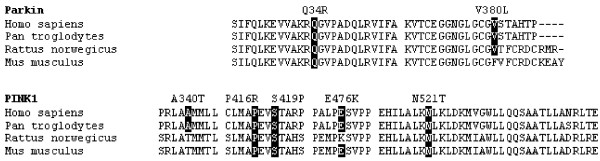
**Multiple sequence alignment of substitutions in the *parkin *and *PINK1 *gene across species using ClustalW version 1.83**. Substitutions are highlighted where conserved and flanked by neighbouring amino acids.

In the *parkin *gene, Gln34Arg (202A>G) was observed in family G (patient II.3) whereas Val380Leu (1139G>C) was observed in family A (patient II.2). Both were in heterozygous state (Segregation patterns figure 1a,c). Multiple sequence alignment showed that Gln34Arg is conserved across mammals and that Val380Leu is limited conserved across mammals (Figure 2a). Both substitutions were predicted by SIFT to be tolerated with a high degree of sequence information (Seq Rep = 0.78).

In the *PINK1 *gene four non-synonymous substitutions were in the serine/threonine kinase domain located to amino acids 156–509 [7]. Substitutions identified in this domain was in exon 5 (A340T), exon 6 (P416R) and exon 7 (S419P and E476K). An additional non-synonymous substitution was located in a large conserved domain 3' of the kinase domain (Figure 2b).

**Figure 3:**
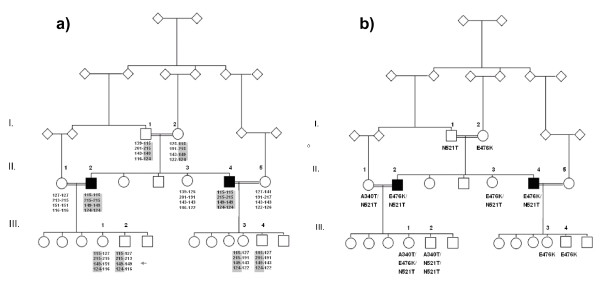
**a-b: Segregation pattern of genetic variation in family F**. Parents of the two affected are 1^st ^cousins. One affected (II.2) is married to his 1^st ^cousin, whereas the other affected (II.4), is married to his 3^rd ^cousin. a Segregation of haplotype and *parkin *exon 4 deletion. Both patients were homozygous for a haplotype of four *parkin *microsatellite markers, and an exon 4 deletion was found to segregate with disease in the family. Gray area denotes haplotype containing exon 4 deletion. b Segregation of substitutions observed in *PINK1*. Both affected are compound heterozygous for the substitutions generally evaluated as common polymorphisms. One of the children, III.2, had a microsatellite mutation or a rarer double recombination (arrow). The fragment lengths of microsatellite markers D6S1599, D6S305, D6S411 and D6S1550 are listed beneath each individual.

The A340T substitution is in a region conserved in primates but not throughout mammals, as illustrated in *Rattus norwegicus *as well as in *Mus musculus *where threonine is the conserved amino acid (Figure 2b). This substitution is found in two families (family F and J, Figure 3b and figure 1f).

In this material, the substitution in the most conserved region is the P416R substitution located in an APE site within the kinase domain, a universally conserved activation segment motif and most likely essential for protein function. This mutation was found in a single family (family G) and was homozygous in both affected individuals (Figure 1c). In close proximity to this universally conserved APE motif is the S419P substitution. This is a putatively important structural change, in a region conserved across mammals. The substitution was homozygous in both affected of one family (family I) and was present together with D391D and N521T both in homozygous state (Figure 1e).

The E476K substitution represents a change in amino acid charge in a residue moderately conserved across mammals. In addition, lysine is the species specific amino acid in the *Rattus norwegicus *form of the *PINK1 *gene, indicating a less severe alteration. Together with N521T this substitutions was found to segregate with disease in one large family (family F) and as heterozygous in members of an additional family (family E, Figure 1b).

The N521T substitution is in exon 8, outside the serine/threonine kinase region, in a conserved region comprising a large sequence at the 3' of the protein. The region is conserved across all mammals but has an unknown function. The N521T substitution identified in this region is the most prevalent in our material and also one of the most common substitution found in cases and family members with early onset parkinsonism, as well as in controls in other studies and is regarded as a polymorphism and not a disease causing mutation [27,31,46]. This variation is present as compound heterozygous in both affected of family F and as compound heterozygous in the only affected of family J (Figure 1f). In family F, the N521T substitution is present with E476K and in family J it is present with A340T. The N521T substitution is also present as homozygous in both affected of family I. However, in family I the substitution is present with S419P, also homozygous and presumably the most prominent substitution of the two. A juvenile onset of parkinsonism was observed in family H with the affected only heterozygous for N521T.

We did not identify any non-synonymous substitutions within family A-D and K which were all from the same region in Jordan (north-Jordan). Family E was the only family from that region making an exception with an E476K non-synonymous substitution in the *PINK1 *gene. The family was identified through a single affected, strictly classifying this as sporadic young-onset parkinsonism.

### Synonymous substitutions

Additionally to the substitutions described above, we observed a synonymous substitution, Val17Val (152C>T), in *parkin *in family G. In *PINK1 *three synonymous substitutions, L63L in exon 1, D391D in exon 6 and L581L in exon 8 were identified. The L63L substitution was present in four affected; family B patient II.1, family D patient II. 2 and family K patient II.1 and patient II.2 (data not shown in figure 1) and has been identified as a polymorphism in a series of studies [45]. The D391D synonymous substitution was present in a homozygous state in family I, in both affected. However, these affected are homozygous for two additional non-synonymous substitutions (figure 1e). The L581L substitution was identified in an unaffected member of family G (member III.1). Predictions using SIFT v.2 strengthened the assumption of the A340S, E476K and N521T substitutions to be tolerable polymorphisms. P416R was predicted not tolerable while S419P was predicted marginally tolerable (p = 0.11, threshold p = 0.05).

Among other noteworthy observations was a tendency of substitutions to accumulate in families. Substitutions are introduced into the pedigree of both family F and G and seem to be highly accumulated in family I. Accumulation of substitutions is a trait expected to appear in highly consanguineous families when clinical symptoms are known or anticipated (selection bias).

### Gene dosage

**Figure 4:**
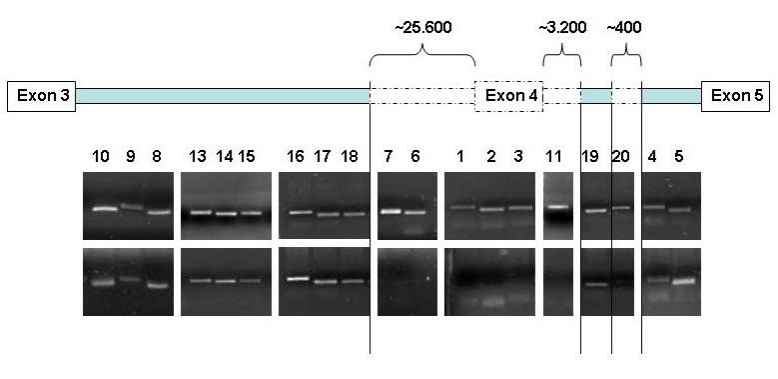
**Mapping of the *parkin *exon 4 deletion in family F**. A composite deletion was identified consisting of one large ~25.600 bp and one small ~400 bp deletion and presence of fragment 19 confirmed by two additional flanking primers. Gel pictures represent control (top row) and patient II.4 of family F (bottom row).

Gene dosage analysis was performed with SALSA MLPA probe set P051 and P052B in affected and/or informative family members of all families. In members of family F, an exon 4 deletion was identified to segregate in a recessively inheritance pattern and homozygous deletions were identified in both affected. Family H had inconsistent control samples and no result was achieved. The extent of the deletion was investigated and a composite exon 4 deletion was observed consisting of one large ~25.600 bp and one small ~400 bp deletion (figure 4).

### Haplotype segregation

DNA samples were genotyped using four microsatellite markers located within the *parkin *gene with the intent to study the inheritance of markers and assess whether if an inherited haplotype could explain the development of PD in some of these families. Homozygous haplotypes were observed only in the two affected in family F. Figure 3a shows the pedigree of family F, where the two patients II.2 and II.4 are homozygous for all markers analysed within the *parkin *gene. We looked at correct phasing to identify any recombination events. Recombination was observed in a healthy individual in family B, offspring II.5 (family tree not shown) and family F, offspring III.2. We also observed recombination events in both the affected offspring (II.1 and II.2) in family K (family tree not shown). The haplotype segregation analysis was used to establish family relations together with results from the AmpFlSTR^® ^profiler^® ^kit. Based on these results, two families were excluded prior to this study. A female cousin in family G was also excluded prior to this study along with two members of family F.

## Discussion

The families included in our study are from regions with a high prevalence of consanguinity, favouring an increased frequency of genetic homozygosity and diseases with recessive inheritance. From the literature, it is well known that genetic variation within the *parkin *gene cause autosomal recessive juvenile parkinsonism (AR-JP) [6,10,17-20,22,40] and is the most common cause of familial parkinsonism known to date [10], while mutations in the *PINK1 *gene are recognized as an increasingly important genetic cause of early-onset parkinsonism. A possible effect of heterozygous mutations in both *parkin *and *PINK1 *have been proposed and a digenic effect have been observed [47]. To this end we identified an exon 4 deletion in *parkin *that was homozygous in both affected (figure 3a) and additional substitutions in *PINK1*, which co-segregated with disease (figure 3b). Some substitutions acknowledged to be common in populations are here discussed according to possible role in disease development.

In our material we identified four non-synonymous substitutions in the kinase domain of *PINK1*, of which two are novel and proposed to be putative pathogenic mutations. The protein kinase domain in *PINK1 *that is located to amino acids 156–509, has a high degree of homology to similar kinases in the Ca^2^+/calmodulin family [7]. Functional studies indicate that mutations in the kinase domain are expected to effect kinase activity as well as substrate binding capability [33].

Affected individuals in these families were either homozygous or compound heterozygous and most substitutions were evaluated to be non-pathogenic polymorphisms but could be discussed in relation to impact of a single hit as well as accumulative effect of substitutions. The P416R and S419P substitutions are novel and homozygous in two affected in each of their respective families (figure 1c, e). Both are in conserved regions of the gene and predictions using SIFT software for the tolerability of substitutions indicates that especially the P416R substitution is of probable functional importance to the protein. The A340T, E476K and N521T substitutions are assumed to be non-pathogenic polymorphisms [27,31,46] and the D391D synonymous substitution is assumed to have no effect [27], although the A340T substitution has been proposed to contribute to risk of development of late-onset PD [57]. Interestingly, in family F, two of these substitutions were found to co-segregate with disease. In the other families with *PINK1 *polymorphisms, a segregation pattern compatible with recessive inheritance could not be excluded, when taken into account the possibility of reduced penetrance in some offspring due to low age.

In family F we observed an E476K substitution and an N521T substitution segregating with disease. Both affected in family F were compound heterozygous for these putative polymorphisms in the *PINK1 *gene. The E476K substitution is in the protein serine/threonine kinase domain of the protein, but only to a small degree conserved across species (figure 2b). The effect of the substitution on the enzymatic activity of *PINK1 *is not known but the substitution is found as the conserved amino acid in several species [27] and are common in controls as well. The N521T substitution is located in a conserved region at the 3' of the protein outside the 156–509 residues proposed as the serine/threonine kinase region. This region is conserved across all mammals and has an unknown function. The N521T substitution identified in this region is the most prevalent in our material and thus far one of the most common substitutions found in cases as well as healthy family members with parkinsonism. In previous studies the N521T substitution has been common and recognised merely as a polymorphism [45,58] and in studies of early-onset PD homozygous incidences have been confirmed in controls and cases equally [27,31].

The exon 4 deletion in the *parkin *gene segregates with disease and explains the clinical manifestations. It is curios thou, how additional substitutions are accumulated in the *PINK1 *gene as revealed in this study, and for this reason the observed substitutions will be discussed.

The A340T substitution introduced into the pedigree of family F was equally found in compound heterozygous state with N521T in family J. Threonine is the conserved amino acid in *Rattus norwegicus *and *Mus musculus *(figure 2) and this suggests the substitution is most likely a polymorphism. However, we cannot completely exclude a possible effect of the A340T polymorphism in combination with supplementary mutations within *PINK1 *or in other genes affecting parkinsonism.

*Parkin *is located in the third most common fragile site, FRA6E, proposed to be involved in several types of cancer, acting as a tumor suppressor gene together with other common fragile site genes such as FHIT (FRA3B ; 3p14.2) and WWOX (FRA16D ;16q23) that are also associated with exon deletions [43,51]. In some cancer cells there are a high frequency of loss-of-heterozygosity (LOH) observed in D6S1599 (*parkin *intron 2) and D6S305 (*parkin *intron 6) [43] as observed in carriers in healthy family members of family F in our study. The mechanism of common fragile sites might indicate some of the mechanism behind the observed *parkin *deletions and associate the formation of deletions with a FRA6E site mechanism. The occurrence of *parkin *mutations could either be independently recurrent de novo mutational events or a geographical spread through founder effect. In general, exon rearrangements are found to be caused by independent de novo mutational events, while point mutations are spread mainly through founder effect [48,49]. The extent of the exon 4 deletion in family F was further mapped and was different from one mapped in a geographically close Turkish family [50].

We found a Gln34Arg substitution previously described in India where it might be the result of a geographic founder effect [52,53]. In India, the substitution was observed in heterozygous state in affected with parkinsonism, but also in older unaffected family members [52].

This might be a dominant negative mutation which contributes to functional variation in the Parkin protein. A functional example may be that the protein still interacts and competes with the wild type protein for the target protein, but lacks functional properties. Therefore, in heterozygous state, this category of mutation might cause a range of phenotypes in response to genetic and environmental influence. Variation in penetrance is discussed for several parkinsonism related mutations. Dominant negative mutations may be a possible explanation of some of the observed heterozygous mutations having a variable "dominant nature". It is different from the loss-of-function mutations generally observed in the *parkin *gene, where a heterozygous state continues to have a normal phenotype.

Abbas and colleagues [17] previously detected a mutation in the same location of exon 2 in two British families, in which a 202-203delAG caused "loss-of-function" of the Parkin protein [17]. The Gln34Arg substitution has not yet been functionally characterized, and its function is therefore still uncertain. The localization to the ubiquitin-like domain suggests it may effect binding to proteasome subunits. The substitution was not observed in the carriers affected sister (patient II.4), who also developed the disease, stating that this substitution is not involved in disease development in both cases. More important, the affected are both carriers of homozygous P416R substitutions which probably cause the clinical symptoms (see discussion on P416R further down).

The Val380Leu mutation has, in previous studies, been found in both healthy and affected individuals. Abbas *et al*. [17] reported that the substitution was found in eleven European families and also 16% of the control subjects and it appear to be common across populations [52,54] and therefore not considered a main cause of disease development. It might, however, alter the Parkin protein and contribution to the pathogenesis of idiopathic PD, as noted in a study by Lucking *et al*. [55], which reported homozygous Val380Leu substitutions to be associated with sporadic PD. Biswas et al [56] commented on this substitution that it might be an association in some populations when ethnically stratified. In our study the substitution was heterozygous in one affected and absent in the carriers brother who also developed young-onset parkinsonism implying that the substitution was not involved in development of disease in both affected family members (figure 1a).

A recessive pattern of inheritance should, in a population with high degree of consanguinity, anticipate the observation of homozygous haplotypes identical by decent (IBD) in the *parkin *gene, as found in family F where the exon 4 deletion and all four microsatellite markers segregated with disease. Further haplotype assessment did not reveal any haplotype that could associate the *parkin *gene with the observed symptoms of young-onset or juvenile parkinsonism across families. Sequencing revealed few substitutions and we were unable to identify specific point mutations in remaining families. Quantitative analysis revealed no exon rearrangements or complete gene deletions/multiplications that could associate the *parkin *gene with the observed clinical symptoms. We confirmed less than expected incidences of *parkin *caused parkinsonism in the population. Multiple sequence alignment and estimation of the tolerability of the observed *parkin *substitutions indicated that they were probably tolerable (Figure 2a). The reported frequency of *parkin *associated parkinsonism was similarly low in populations of India where the Gln34Arg substitution was previously observed [52,53].

In *PINK1*, the novel P416R substitution was identified in family G, in a highly conserved motif of the activation segment in the serine/threonine kinase domain [33]. The substitution was homozygous in both affected members of the family and were the centre amino acid of an APE motif conserved in orthologous sequences and in paralogous protein kinases [33]. Located in a universally conserved functional motif the substitution is likely to affect kinase activity.

The S419P substitution was identified in family I. Two non-synonymous substitutions and one synonymous substitution, all in homozygous state, were identified in both affected members of the family (figure 2). Located in the serine/threonine kinase domain the S419P substitution was the most conserved alteration within the material.

There were no obvious differences between the ages at onset amongst putative *parkin *and *PINK1 *mutation carriers as compared to non-carriers (Table 1). However, there are similarities regarding age at onset within affected of the same family. For affected carrying P416R, the only substitution to be in a universally highly conserved motif, an earlier age at onset was seen in both affected, as compared to family I carrying three homozygous substitutions, amongst them the S419P substitution. These findings indicate a severe function of the P416R mutation, which could represent a total loss-of-function mutation. Otherwise, the observed variations in age at onset could generally be due to interactions of additional environmental or genetic factors such as accumulation of substitutions causing gradual alterations in protein structure. In light of the SIFT predictions, assessment of the tolerability of the S419P substitution is dependent on further functional analysis.

In some families an accumulation of substitutions are observed. In family F both deletion in *parkin *and substitutions in *PINK1 *co-segregate with disease, and a carrier of A340T and N521T is married in. In family G a carrier of N521T is married in to the family through a healthy family member. In family I, the accumulation of substitutions in the two affected, the only two persons available for analysis, are notable. The possibility of a LOH for this region was excluded using the gene and exon dosage SALSA MLPA kit. Accumulation of substitutions should suggest some selective bias based on knowledge of disease in families and therefore suggest a role of these substitutions in disease or in association to disease.

## Conclusion

The role of digenic inheritance of *parkin *and *PINK1*, impact of a single mutation hit and accumulation load of gene polymorphisms are unclear. We observed a three generation family with an exon 4 deletion in the *parkin *gene and substitutions in the *PINK1 *gene co-segregating with disease indicating a potential digenic effect.

We identified two novel substitutions in the *PINK1 *serine/threonine kinase domain with the P416R as probable and the S419P as possible putative pathogenic mutation and the genetic cause of young-onset parkinsonism in two families.

## Competing interests

The authors declare that they have no competing interests.

## Authors' contributions

RM contributed to writing of manuscript, laboratory work and guidance and interpretation of results. Co-authors AS, SS and GLN performed laboratory work with interpretation of results and contributed with the manuscript. KH was the neurologist assessing the patient's diagnoses and HAZ contributed with laboratory work and interpretation of material in Jordan where MKN contributed to conception and design and was project co-ordinator. HK contributed to conception and design and was project co-ordinator in Norway.

## Pre-publication history

The pre-publication history for this paper can be accessed here:


